# Supplementary material for: ProteinShader: illustrative rendering of macromolecules
Source: BMC Struct Biol. 2009 Mar 30;9:19. doi: 10.1186/1472-6807-9-19 (PMC2672931; doi:10.1186/1472-6807-9-19)
Supplement: Additional file 1 — ProteinShader program without source code. This compressed file contains the complete ProteinShader program including associated libraries, but no source code. A README.txt file gives an overview of the ProteinShader distribution, and the index.html file in the help subdirectory has directions on getting started with the program as well as a set of tutorials. [file 1472-6807-9-19-S1.zip › ProteinShader-beta-0_9_4-binary/help/api/org/proteinshader/gui/components/controlpanel/package-summary.html]

org.proteinshader.gui.components.controlpanel (ProteinShader API)


|  |  |  |
| --- | --- | --- |
| **Overview** \| **Package** \| Class \| **Use** \| **Tree** \| **Deprecated** \| **Index** \| **Help** | |  |
| **PREV PACKAGE**   **NEXT PACKAGE** | **FRAMES**    **NO FRAMES**     **All Classes** |  |


---

## Package org.proteinshader.gui.components.controlpanel

Holds the JPanels used in the right-side retractable control panel of the GUI.

**See:**
  
          **Description**

| **Class Summary** | |
| --- | --- |
| **AntialiasingPanel** | This control panel allows the user to turn antialiasing (edge smoothing) effects on or off. |
| **AtomColorPanel** | This control panel allows the user to modify the color of Atoms. |
| **AtomScalePanel** | This control panel allows the user to modify the scale for the radii of Atoms in Space-Filling or Balls-and-Sticks style displays. |
| **AtomVisibilityPanel** | This control panel allows the user to modify the visibility status and alpha value of Atoms. |
| **CartoonColorPanel** | This control panel allows the user to modify the color of Segments. |
| **CartoonSideChainsPanel** | This control panel allows the user to display side chain Atoms and Bonds while in a Cartoon-style display. |
| **CartoonVisibilityPanel** | This control panel allows the user to modify the visibility status and alpha value of Segments. |
| **ColorPanel** | This control panel allows the user to modify the color of Drawable objects. |
| **ControlPanel** | Presents the SelectorPanel and ModifierPanel. |
| **DecorationsPanel** | This control panel allows the user to select a decoration type (Plain, Text Labels, or Halftoning) for Segments of a tube or ribbon. |
| **ModifierPanel** | Presents the user with a choice of several control panels for changing color, visiblity, scale, motion, and other features of the display. |
| **MotionPanel** | This control panel allows the user to rotate the image or move the camera. |
| **OptimizePanel** | This control panel allows the user to optimize the display for image quality or rendering speed. |
| **RadioPanel** | Presents a common set of radio buttons and menus that are used by other panels (color, visibility, and scale panels). |
| **SelectorPanel** | Provides a panel with menus and lists for selecting a Structure's Models, Chains, Residues, Atoms, Helices, and BetaStrands. |
| **VisibilityPanel** | This control panel allows the user to modify the visibility status and alpha value of Drawable objects. |

| **Enum Summary** | |
| --- | --- |
| **RadioPanel.Type** | There are two slightly different versions of the RadioPanel. |

## Package org.proteinshader.gui.components.controlpanel Description

Holds the JPanels used in the right-side retractable control panel of the GUI.
